# Beyond kindness: a proposal for the flourishing of science and scientists alike

**DOI:** 10.1098/rsos.230728

**Published:** 2023-11-22

**Authors:** Frank Schumann, Mareike Smolka, Zoltan Dienes, Annika Lübbert, Wolfgang Lukas, Mary Gehring Rees, Enrico Fucci, Marieke van Vugt

**Affiliations:** ^1^ Laboratoire des systèmes perceptifs, Département d’études cognitives, École normale supérieure, PSL University, CNRS, 75005 Paris, France; ^2^ Sorbonne Université, INSERM, CNRS, Institut de la Vision, 75012 Paris, France; ^3^ Université de Paris, CNRS, Integrative Neuroscience and Cognition Center, 75006 Paris, France; ^4^ Knowledge, Technology and Innovation, Wageningen University and Research, Wageningen, The Netherlands; ^5^ Human Technology Center, RWTH Aachen University, Aachen, Germany; ^6^ School of Psychology, University of Sussex, Falmer, Brighton, UK; ^7^ Independent Researcher, Hamburg, Germany; ^8^ Institute for Globally Distributed Open Research and Education (IGDORE), Graz, Austria; ^9^ Independent Researcher, Houston, TX, USA; ^10^ Institute for Globally Distributed Open Research and Education (IGDORE), Gothenburg, Sweden; ^11^ Bernoulli Institute of Mathematics, Computer Science and Artificial Intelligence, University of Groningen, Groningen, The Netherlands

**Keywords:** flourishing, science policy, open science, ethics

## Abstract

We argue that many of the crises currently afflicting science can be associated with a present failure of science to sufficiently embody its own values. Here, we propose a response beyond mere crisis resolution based on the observation that an ethical framework of flourishing derived from the Buddhist tradition aligns surprisingly well with the values of science itself. This alignment, we argue, suggests a recasting of science from a competitively managed activity of knowledge production to a collaboratively organized moral practice that puts kindness and sharing at its core. We end by examining how Flourishing Science could be embodied in academic practice, from individual to organizational levels, and how that could help to arrive at a flourishing of scientists and science alike.

## 1. Introduction

Science is a remarkably productive human enterprise, yet recent revelations of multiple crises underscore a pressing need for reform and improvement. These problems manifest in compromising the quality of science as well as the rate with which science can generate knowledge, for example through ethically dubious research practices [1], a replicability crisis [2] or innovation biases [3,4].^1^ There is also a loss of talent [5] as ever more researchers are leaving science when facing mental health concerns, such as anxiety, chronic stress and exhaustion [6], as they struggle with a culture of precarious and short-term working conditions [7–9], power-abuse, exploitation, bullying and harassment [10–13].

This opinion piece argues that an important common cause of all these problems is a present failure of science to sufficiently embody its values.^2^ One prominent cause for this failure of embodiment, in turn, is the large-scale managerial transformation of the academic enterprise [17,18], leading towards an unsustainable [19] hyper-competition that has promoted (too) much ego-centric behaviour and (too) little of the moral and collaborative behaviour required for ‘doing good science'. In addition, societal inequalities have expressed themselves detrimentally within and through science and academia as well [20–22]. Policy-based value-centred initiatives to reverse some of these impediments have faced difficulties in putting the respective value-centred frameworks into practice (e.g. [23]), suggesting a gap between understanding a value and endorsing it in everyday life actions [24].

Here, responding to calls for a ‘kinder research culture' [25,26], we suggest an integrated value reform that builds on ideas of secular ethics, such as promoted by the Dalai Lama [27] or the compassion-based curriculum at Emory University [28]. Going beyond kindness, however, we propose a universal ethical framework that supports *flourishing,* known in the Buddhist tradition as the *Brahmaviharas* or the *Four Immeasurables* [29,30] (table 1). The *Brahmaviharas* refer to a beneficial attitude or ‘dwelling place' from which to observe and act. The boundless or ‘immeasurable' scope refers to the idea that they support flourishing universally, in any situation, at multiple levels of social organization, towards all parts of oneself as well as others.

**Table 1.** Overview of values that support flourishing.

values supporting flourishing	description
impartiality	quality of not being biased or prejudiced
solidarity	benevolent attitude towards others
compassion	empathetic concern for the suffering of others
empathetic joy	delight in the achievement of others

We argue that these four immeasurable values, viewed or reinterpreted in a certain way, match remarkably well with the values and attitudes of science itself. We show that an aspect of each value is core to how science functions qua science, that is, as a tradition that enables the growth of knowledge. Furthermore, their underlying framework of flourishing acts as an antidote to the disruptive aspects of self-focused attitudes and behaviours as well as the inequalities that appear to evoke much of the current problems. Thus, more broadly, we offer flourishing as an alternative to the contemporary ego-focused competitive model of organizing science. Flourishing Science, instead, focuses on enabling people to work together well, and is thereby well aligned with core values of science itself. We argue that such a foundational re-alignment of how the process of science is viewed and approached can act as an orienting guide for iteratively finding and revising solutions to the current problems of academia. Although intention is not action, having the right framework from which to act may in fact still be more critical than any particular solution proposed, such as advocating kindness or Open Science practices. Although we focus here on the natural sciences, a Flourishing Science framework could equally support flourishing in other academic disciplines, such as the social sciences and the humanities.

## 2. Values for a flourishing of science

The Oxford English Dictionary defines flourishing as to ‘grow or develop in a healthy or vigorous way, especially as the result of a particularly congenial environment'.^3^ Before describing how the *Four Immeasurables* could aid the flourishing of science in the next section, we first define them, aligning a traditional view of each value with a tentative interpretation within the domain of science.

***Impartiality****,* or equanimity, is the virtue and practice of remaining non-attached, balanced and even-minded without being biased or prejudiced. In this way, impartiality is consistent with a core epistemic value and attitude of science, namely, to see theory, predictions, data and their connections as having a reality apart from us and our wants, desires and beliefs.^4^ Making those connections as objective and as public as possible, and impartially following wherever the results lead, is a fundamental part of what makes science what it is. Another aspect of impartiality is that scientists also need to take other perspectives seriously, ranging from the strongest version of an opposing argument to the perspectives held by stakeholders. Importantly, impartiality is neither indifferent nor cold. It is consistent with being intensely curious or motivated to discover the properties of theories and data, precisely because there is something that is beyond oneself to be explored.

***Solidarity****,* or loving-kindness, is the virtue and intention of a benevolent and supportive attitude towards everyone, including oneself. Science progresses only because researchers openly provide knowledge and solutions that others can build upon [33]. Solidarity relies on recognizing mutual dependence, and thus on humility, mutual support and collaboration towards common goals.

***Compassion****,* going beyond solidarity, is the virtue of holding an empathetic concern for the suffering of others, whereby one notices, feels and acts to ease and transform that suffering and its causes. Science involves problems no one person alone can solve; hence, we should be concerned about the problems of others, scientists and non-scientists alike.

***Empathetic joy*** means to practice taking delight in the achievement of others. It suggests that we can be motivated by solving problems, and by doing so together, rather than by maximizing individualist metrics, such as an h-index, realizing it is only because others succeed that we can build on those successes. Cultivating empathetic joy helps academics appreciate and support not only their own but also other perspectives and successes.

## 3. Initiatives and recommendations for a flourishing of science

**Figure 1.**
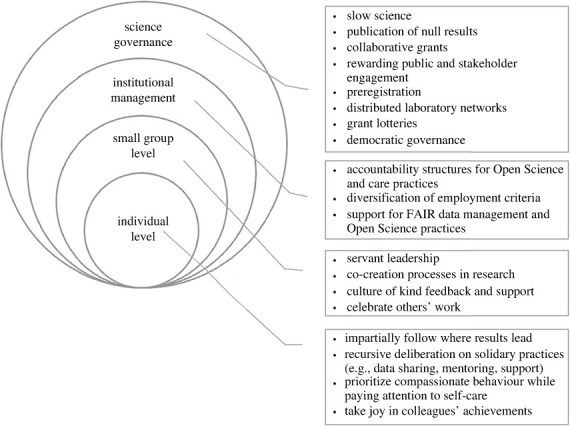
Suggestions for putting a Flourishing Science framework into practice.

We introduce the *Four Immeasurables* so that they may serve as qualities to bring about personal and collective flourishing in academia. The boundlessness of these values (hence their metaphoric ‘immeasurability') sees their embodiment both with the individuals putting them into practice as well as with conducive organizational structures and support. That is, the boundless scope of the *Four Immeasurables* recognizes that individual action occurs within larger embeddings and thus encompasses both individual and systemic levels. This means, first, that the impact of individuals' ethical actions depends on the institutional and wider political conditions under which they operate. And second, that for individuals to be rewarded and safe when engaging in ethical action, suitable structures are required at the institutional level. By highlighting this boundless nature, we aim to raise awareness and safeguard against individualizing systemic issues: one should not divert attention from systemic levels by placing the onus only on individuals, nor vice versa, but consider both personal and institutional responsibility. In this section, we begin to explore what an implementation could look like (figure 1). We do so separately for the individual and the institutional levels for clarity of exposition, while recognizing their tight interrelatedness.

### 3.1. Individual and small group levels

The Buddhist tradition considers the *Four Immeasurables* or the *Brahmaviharas* as virtues that form our ‘best home', a ‘dwelling place’ that is beneficial to our observations and actions. *Brahma* means the ‘supreme' or best, while *vihara* means dwelling or home. A common recommendation for their practice is that they are not simply abstract values to be acquired or preserved, but contextual, situational values to be continuously experienced, sensed and reflected upon to cultivate a ground from which desired things can emerge, in one's own situation and life [34]. Compassion and kindness, for example, are not merely about saying the right words, just like jazz is not merely about playing the right notes [35]. In the following, we seek to begin a reflection on cultivating the *Four Immeasurables* in the context of science on individual and small group levels.

#### 3.1.1. Impartiality

Can be cultivated by establishing an attitude of non-attachment and intellectual humility that impartially considers and questions different ways of seeing and understanding the world, including and beyond one's own. This attitude can be developed, individually and collectively, by impartially focusing on wherever our results and data lead rather than on what we want (or may need) them to be. It can also be cultivated in co-creation processes that involve diverse research contributors, ranging from technicians, junior and senior researchers, to research participants. We have been trying to put this into practice. For example, the work of Schumann & O'Regan [36] on sensory augmentation derives from an openness to follow results that did not align with their own theory, re-visiting and reaching a fresh perspective on assumptions and theoretical tools. In the work by van Vugt *et al*. [37] on monastic debate, Tibetan monks are not only enlisted as participants who perform a predetermined task, but also as active co-researchers whose views are incorporated in designing, executing and interpreting the research.

On the individual level, one can practice impartiality by reflecting on aspects that are conducive to promoting our attention to these attitudes and qualities, such as remembering that science is a long-term project that transcends a lifetime and that a scientific claim thus is not yours, and not you. Or that, hence, in presenting a claim, or criticizing one, it is advisable to not conflate claims with people. Where possible, say not ‘Your claim …', nor ‘My theory …', but ‘The claim that … is consistent with this argument … but not this …'. This allows one's interlocutor to consider arguments in themselves without being defensive. Such reflections can cultivate an inner ground from which we can construct an atmosphere of exploring the objective properties of claims in which people are jointly curious about what might be found. In this, we may catch ourselves when we are about to say, ‘Your theory …', and check ourselves as we launch into criticism. A beneficial attitude here can be to approach such practice with a sense of renewal that recognizes the habitual nature of our awareness and the resulting need to begin again and again from good intention without too many regrets for having made a mistake or for time seemingly squandered. In this way, the *Four Immeasurables* can be practiced distinctively, but they can also be taken as vehicles for each other. Solidarity, for example, is supported by our capacity for equanimity or impartiality [34].

#### 3.1.2. Solidarity

Suggests that science thrives when researchers can support and collaborate with each other, sharing ideas, data and technologies^5^ rather than secure competitive advantages required for climbing the career ladder. To practice solidarity, we encourage contemplation and deliberation on questions, such as: How do we help colleagues with tasks, duties, and difficulties they face? To what extent do we share data, expertise and know-how as well as experience and enthusiasm? How do we support those at lower levels by means of mentoring and sponsoring? (How) do we support others when providing critique, for example, after a talk or in a review of their work? Here we encourage reflection on and practice of game theorist Rapoport's rules for productive discourse. In Dennett's reformulation, when composing a critical commentary towards another ‘(1) You should attempt to re-express your target's position so clearly, vividly, and fairly that your target says, **‘**Thanks, I wish I'd thought of putting it that way'. (2): You should list any points of agreement (especially if they are not matters of general or widespread agreement). (3) You should mention anything you have learned from your target. (4) Only then are you permitted to say so much as a word of rebuttal or criticism' ([38], p. 33).^6^

Solidarity does not mean that we should give in to everybody and to every demand, but that we consider our work as part of a bigger fabric of knowledge and ideas. In this light, we can also ask ourselves: What do I truly strive for, and how can I find the support that I need? At the group level, an interesting approach from the management literature in this direction is ‘servant leadership' [39], in which leaders foremost serve to develop their teams by asking how its individuals can thrive and grow. The boundless scope of the *Four Immeasurables* means that they are applied to all scientists, irrespective of status, gender or ethnicity, and within one's peer group, but also within the larger system. At the group level, such a culture of care can mitigate or prevent bullying [11]. It can also be organized outside academic institutions in informal grass roots gatherings, a form of giving advice and mutual support that has been practiced by high-level scientists around the world [40]. More formal initiatives outside academia, such as the Academic Parity Movement^7^, can provide external support for victims of harassment and help raise awareness about its occurrence. Such initiatives, however, should not lead us to overlook the distinct power that already close bystanders have to intervene [11,41]. Further, initiatives like the Neuromatch^8^ movement can work towards equitable participation and mutual support in scientific research by providing open source teaching and connecting people in virtual summer schools and conferences [42].

#### 3.1.3. Compassion

Can be practiced by holding an empathetic concern towards all, within and outside science. This requires the skill to recognize suffering without getting overwhelmed by it or pushing it away, and to act to ease or alleviate its cause. To enact compassion, we suggest deliberate contemplation and skillful action towards suffering at all levels, small and large: towards scientists struggling to understand a specific phenomenon or idea, by approaching feedback in talks and reviews as an opportunity to help improve others' work; towards problems of non-scientists that may be alleviated through scientific results and tools; towards working conditions that do not meet individual needs; towards those who feel they have to employ questionable practices such as p-hacking to be competitive; towards seeing mistakes as an opportunity to learn, support, and to implement work routines that provide collective safeguards (e.g. open data, open materials, pre-registered analyses). To support solidarity with a compassionate way of composing critique, construct the strongest version of your discussant's position, to see the strength of it. Make sure any weakness is a weakness of the strongest position. Then consider how it could be addressed. Compassion, in other words, is a way of creating kind and supportive environments where scientists can flourish in producing and questioning theories with openness and dignity.

#### 3.1.4. Empathetic joy

Being happy for someone else's success, is a virtue that can counteract jealousy. This form of joy can, traditionally, be practiced by reflecting on phrases, such as ‘may I be happy, may you be happy', or by reflecting on assumptions, such as if revengefulness really makes us strong [34]. With regards to science, we propose joy can be actively promoted by seeking to understand what it is that others aim to understand, why it is valuable, and what is difficult about it, such that their successes become meaningful. We can also consciously make practical efforts of rejoicing in communal celebrations, especially of small achievements, such as solving a problem with one's experimental set-up, having an idea for a required control, or the completion of data collection. Published articles or outreach activities, on the other hand, can be publicly displayed in universities and research institutes to generate awareness and appreciation of the work of colleagues. Crucially, again, the boundless scope of the *Four Immeasurables* implies an impartial, unbiased celebration of successes for all, regardless of gender, position or ethnicity.

### 3.2. Science governance and institutional management

The boundless scope of the *Four Immeasurables* also means that they support flourishing via multiple levels. Individual and group actions are both empowered and constrained by the organizations in which they are embedded. As such, science governance and management structures that allow and promote ethical action are equally critical for the flourishing of science.

#### 3.2.1. Impartiality

To empower impartiality and promote individual researchers' engagement with relevant phenomena in the most unbiased way, the academic system could provide support structures in multiple ways: for example, enable the tenets of ‘slow science' [43,44], encourage reflection on positionality, reward publication of methodologically rigorous studies regardless of results, give well-developed projects more equal chances for funding, and institute more democratic governance structures. Slow science proposes to think in longer time horizons and with the larger value of science in mind. It sees science as an ‘astonishing human effort, which transcends an individual's lifetime', where researchers ‘have reason to believe that science continuously improves our models of the world'. Thus a ‘farsighted vision is necessary to create and test big theories, regardless of obstacles' [44].^9^ We believe that such a vision must crucially include supportive systems that empower scientists to do so. Decelerating the academic work pace with long-term support gives researchers critical time for impartial reflection so that biases and prejudices can be recognized and overcome, by contemplating and questioning their own and their colleagues' work or the perspectives of stakeholders via public engagement.

Researchers and reviewers could be encouraged in job applications and evaluations to reflect on assumptions and interests that may stem from their disciplinary background, identity, howsoever they wish to define it, and personal beliefs they judge relevant. Note that boundless equanimity however means *striving to decentre* from the constraints of particular identities, a practice which is at odds with constantly defining oneself by fixed identities (e.g. race, gender or sexuality). That is, a Buddhist approach to social inequalities does not align with some other approaches that might be used to address social inequality, namely approaches urging constant strong identifications.

Furthermore, researchers could develop measures such as those suggested by Open Science initiatives, including the preregistration of research hypotheses [47] or Bayesian model comparison making assumptions explicit [48]. Rewarding the publication of null results fosters the study of what is scientifically most interesting rather than easiest to publish. The use of lotteries to select grant proposals that show merit and pass quality standards can enable a more impartial distribution of funding. Lotteries can introduce a degree of randomness either in pre-lotteries at early stages or as ‘tiebreakers' at final stages of the grant evaluation process. A degree of randomness can counteract biases, ranging from innovation bias (favouring familiar over highly novel ideas) and biases due to sexism, racism, ableism and equality issues to accumulative advantages through the Matthew effect.^10^ Such lotteries could also enable riskier, more innovative and interdisciplinary proposals, especially when competing for funding under conditions of high path dependencies that incentivize predictable low risk projects feasible within the typically short project-based time frames [3,4].

Lastly, democratic governance, such as the use of citizens assemblies, and open democracy more generally [49], can impartially allow all citizens of an institution to engage in decision-making in any important decision that affects an institution [50].

#### 3.2.2. Solidarity

To facilitate solidarity via science management, we propose to work on system structures that support and adequately reward collaborative achievements and collaboration more generally. To reward collaborative achievements, one could, for instance, encourage distributed laboratory networks, such as the Psychological Science Accelerator^11^ or Registered Reports, that promote collaboration rather than conflict between reviewers, authors and editors. Similarly, one could move from individual to collaborative grants, such as the ERC synergy grant [51] and restructure the concept of authorship to reflect such collaborative practice. Scientific prizes could be awarded not only to individual researchers, celebrating their seemingly independent achievements, but also to those who support them. To mitigate academic bullying, organizational structures could discourage rather than reinforce egoistic behaviours, in line with the four immeasurable values that we here propose. Such measures encompass organizational justice and transparency [52], including clear and fair structures for reporting and investigation. These could follow a transparent code of conduct [53], allow reporting without fear of reprisal [54,55], and show that bullying has consequences even for those in high positions [10]. Furthermore, the bulk of resources in academia are currently in possession of the Global North. Open Science practices could allow for more location-independent work that may foster a higher dispersion of resources and expertise around the globe. The Ronin Institute^12^ and the Institute for Globally Distributed Open Research and Education (IGDORE)^13^ are examples of promoting this way of working.

#### 3.2.3. Compassion

To promote compassion, the governance structure of institutions should reflect the values to be enacted by their members. Authoritarian control in a purely top-down managerial governance structure is unlikely to promote the mutual respect and concern required for compassionate collaboration on difficult problems, nor does it boost morale. Open democratic processes that engage researchers with real decision-making power would allow the culture of governance to more closely mirror the culture of science itself that we are promoting (see also [18,50]). As this will take a long time to realize, we suggest that even within existing suboptimal managerial governance structures, compassionate behaviour can and should be rewarded. One possibility is to include in researchers' evaluations how they practice sharing (e.g. of data and findings) and how they create a culture of caring [56].

#### 3.2.4. Empathetic joy

Joy can be promoted through the alleviation of hyper-competition, which makes it easier for individual researchers to be happy for and to support the successes of others. Exemplary actions include basing assessments of researchers in richer evaluation criteria than primarily the amount of funding they acquired and the number of yearly publications they achieved, following, for example, the DORA declaration.^14^ Similarly, as Frith [44] proposes, the quality of collaborative academic work may increase if the ways in which one takes delight in giving others support becomes relevant for employment. Furthermore, new funding instruments could distribute small grants across a number of research projects rather than investing large grants in single individuals [57].

## 4. Conclusion

In short, we support calls for a kinder science by pointing to a close relation between traditional values that make groups work together well and the values of science itself. On individual and small group levels, we have given examples of how one could embody these values in academic work, which we argue can foster impartial (joint) insights and collaboration. We have also given examples of how this could be promoted on institutional and science governance levels. Beyond advocating for merely a kinder or more open science, however, our proposal suggests Flourishing Science as a new framework for a fundamental shift in the activity of knowledge production: embracing collaborative values, such as the *Four Immeasurables*, can transform the organization of science from a competitive and managerial activity towards a more collaborative and democratically organized moral practice. We believe that science would greatly benefit from actualizing values that allow people to flourish in any social setting: impartiality, solidarity, compassion and joy. While these values are intrinsically latent or inherent in science, they need to be brought more to the forefront. This would also yield many principles of Open Science without the aggressiveness and glee in shaming with which Open Science is sometimes associated. Putting the *Four Immeasurables* into practice in this article ourselves, we emphasize that we offer these contemplations as prompts, not as guidelines, for much-needed reflection and effort towards a greater flourishing of researchers, science, and academia at large.

## Data Availability

This article has no additional data.

## Footnotes

1 Innovation bias refers to a systematic tendency of grant reviewers to favour traditional or familiar projects at the expense of more novel or groundbreaking ones.

2 While literature in the social studies of science has emphasized the multiplicity of scientific values, which have been shown to change over time and across academic disciplines [14,15], here we argue from a natural science position that natural science entails values akin to the Mertonian norms of disinterestedness, communalism, universalism and organized skepticism [16], and that they can be matched with the *Four Immeasurables.*

3 https://dictionary.cambridge.org/dictionary/english/flourish.

4 In the light of the different disciplinary backgrounds of the authors, we emphasize that this statement describes a natural science attitude. Research in the social studies of science sheds light on the entanglements between scientific theories, predictions and data, on the one hand, and socio-cultural practices, believes and motivations on the other (e.g. [31,32]).

5 Sharing ideas, data and technologies can conflict with other values, for instance, with study participants' privacy. Openness and solidarity thus need to be weighed against other values in research practice.

6 Dennett [38] provides a nuanced discussion as well as experience from putting the rules in practice.

7 https://paritymovement.org/.

8 https://neuromatch.io/.

9 A long-term perspective finds support in the history of science, which indicates that researchers and scholars had original ideas and farsighted visions that have proven influential on scientific or societal levels in the generations to come but were largely ignored by their contemporaries. For example, it took decades for the insights of Ludwik Fleck about the origin and growth of scientific facts or for Alain Prochaintz's insights about synaptic plasticity and the growth of neurons in adult life to become as widely understood and influential as they are today [45,46].

10 The Matthew effect refers to the idea that individuals who already have advantages (e.g. recognition) tend to receive more benefits, leading to a cumulative advantage in situations such as grant evaluations.

11 https://psysciacc.org/.

12 https://ronininstitute.org/.

13 https://igdore.org/.

14 https://sfdora.org/.

## References

[1] Joynson C, Leyser O. 2015 The culture of scientific research. F1000Research **4**, 66. (10.12688/f1000research.6163.1) 25866623 PMC4376168

[2] Nosek BA et al. 2015 Promoting an open research culture. Science **348**, 1422-1425. (10.1126/science.aab2374) 26113702 PMC4550299

[3] Langer JS. 2012 Enabling scientific innovation. Science **338**, 171. (10.1126/science.1230947) 23066044

[4] Luebber F, Krach S, Martinez Mateo M, Paulus FM, Rademacher L, Rahal R-M, Specht J. 2023 Rethink funding by putting the lottery first. Nat. Hum. Behav. **7**, Article 7. (10.1038/s41562-023-01649-y) 37349356

[5] Gewin V. 2022 Has the ‘great resignation’ hit academia? Nature **606**, 211-213. (10.1038/d41586-022-01512-6) 35641675

[6] Loissel E. 2020 Mental health in academia: shedding light on those who provide support. ELife **9**, e64739. (10.7554/eLife.64739) 33226339 PMC7682984

[7] Bahr A, Blume C, Eichhorn K, Kubon S. 2021 With #IchBinHanna, German academia protests against a law that forces researchers out. Nat. Hum. Behav. **5**, 1114-1115. (10.1038/s41562-021-01178-6) 34341553

[8] Maher B, Anfres MS. 2016 Young scientists under pressure: what the data show. Nature **538**, 444. (10.1038/538444a) 27786225

[9] Rahal R-M et al. 2023 Quality research needs good working conditions. Nat. Hum. Behav. **7**, 164-167. (10.1038/s41562-022-01508-2) 36755134

[10] Else H. 2018 Does science have a bullying problem? Nature **563**, Article 7733. (10.1038/d41586-018-07532-5) 30487619

[11] Naezer M, van den Brink M, Benschop Y. 2021 Harassment in Dutch academia Exploring manifestations, facilitating factors, effects and solutions. See https://www.lnvh.nl/uploads/moxiemanager/LNVH_rapport__lsquo_Harassment_in_Dutch_academia__Exploring_manifestations__facilitating_factors__effects_and_solutions_rsquo_.pdf (accessed on 23 September 2023).

[12] Moss S, Mahmoudi M. 2021 STEM the bullying: an empirical investigation of abusive supervision in academic science. SSRN Electr. J. **40**, 1-11. (10.2139/ssrn.3850784) PMC8433114 34527894

[13] Täuber S, Mahmoudi M. 2022 How bullying becomes a career tool. Nat. Hum. Behav. **6**, 475. (10.1038/s41562-022-01311-z) 35132170

[14] Daston L, Galison P. 2010 Objectivity. Princeton, NJ: Princeton University Press.

[15] Smolka M. 2022 Ethics in action: multi-sited engaged ethnography on valuation work in contemplative science. PhD Dissertation, Maastricht University. (10.26481/dis.20221011ms)

[16] Merton RK. 1973 [1942] The sociology of science: theoretical and empirical investigations. Chicago, IL: University of Chicago Press.

[17] Münch R. 2014 Academic capitalism: universities in the global struggle for excellence. London, UK: Routledge.

[18] Brette R. 2022 Le modèle managérial de la recherche. Critique et alternative. Médecine/Sciences **38**, Article 1. (10.1051/medsci/2021247) 35060892

[19] Alberts B, Kirschner MW, Tilghman S, Varmus H. 2014 Rescuing US biomedical research from its systemic flaws. Proc. Natl Acad. Sci. USA **111**, 5773-5777. (10.1073/pnas.1404402111) 24733905 PMC4000813

[20] Diele-Viegas LM, Cordeiro TEF, Emmerich T, Hipólito J, Queiroz-Souza C, Sousa F, Vançan AC, Leite L. 2021 Potential solutions for discrimination in STEM. Nat. Hum. Behav. **5**, 672-674. (10.1038/s41562-021-01104-w) 33875839

[21] PLOS Biology Staff Editors. 2021 How PLOS Biology aims to foster diversity, equity and inclusion in science. PLoS Biol. **19**, e3001102. (10.1371/journal.pbio.3001102) 33690631 PMC7942977

[22] Tzovara A et al. 2021 Embracing diversity and inclusivity in an academic setting: insights from the organization for human brain mapping. Neuroimage **229**, 117742. (10.1016/j.neuroimage.2021.117742) 33454405

[23] Naddaf M. 2023 ‘Intention is not action’: brain-research centre steps up quest for equality. Nature **25**, 272-279. (10.1038/d41586-023-01425-y) 37121928

[24] Verplanken B, Orbell S. 2022 Attitudes, habits and behaviour change. Annu. Rev. Psychol. **73**, 327-352. (10.1146/annurev-psych-020821-011744) 34587780

[25] Anonymous. 2019 A kinder research culture is possible. Nature **574**, 5-6. (10.1038/d41586-019-02951-4) 31576053

[26] Russell S, Foulkes I. 2019 Embedding a positive research culture that fosters innovation. Nat. Rev. **19**, 241-242. 10.1038/s41568-019-0127-7 30816349

[27] Gyatso T, Norman A. 2010 Ancient wisdom, modern world: ethics for the new millennium. London, UK: Abacus.

[28] Ash M, Harrison T, Pinto M, DiClemente R, Negi LT. 2021 A model for cognitively-based compassion training: theoretical underpinnings and proposed mechanisms. Soc. Theory Health **19**, 43-67. (10.1057/s41285-019-00124-x)

[29] Aronson HB. 1999 Love and sympathy in Theravāda Buddhism. Delhi: Motilal Banarsidass.

[30] Klein AC. 2014 The four immeasurables. How to deepen equanimity, love, compassion, and joy. *Tricycle.* Accessed on 1 December 2022. See https://tricycle.org/magazine/four-immeasurables/

[31] Latour B, Woolgar S. 1986 Laboratory life: the construction of scientific facts. Princeton, NJ: Princeton University Press.

[32] Traweek S. 1988 Beamtimes and lifetimes. The world of high energy physicists. Cambridge, MA: Harvard University Press.

[33] Xu F, Wu L, Evans L. 2022 Flat teams drive scientific innovation. Proc. Natl Acad. Sci. USA **119**, e2200927119. (10.1073/pnas.2200927119) 35658076 PMC9191666

[34] Salzberg S. 2014 *Sharon Salzberg—MettaHour—Ep. 04—The Brahma Viharas.*. See https://soundcloud.com/beherenownetwork/sharon-salzberg-metta-hour-episode-04-thebrahma-viharas (accessed on April 23, 2023).

[35] Torrance S, Schumann F. 2019 The spur of the moment: what jazz improvisation tells cognitive science. AI & Society **34**, 251-268. (10.1007/s00146-018-0838-4)

[36] Schumann F, O'Regan JK. 2017 Sensory augmentation: integration of an auditory compass signal into human perception of space. Sci. Rep. **14**, 42197. (10.1038/srep42197) PMC5307328 28195187

[37] van Vugt MK et al. 2020 Inter-brain synchronization in the practice of Tibetan monastic debate. Mindfulness **11**, 1105-1119. (10.1007/s12671-020-01338-1)

[38] Dennett DC. 2013 Intuition pumps and other tools for thinking. New York, NY: W. W. Norton & Company.

[39] Nathan E, Robin M, Sendjaya S, van Dierendonck D, Liden RC. 2019 Servant leadership: a systemic review and call for future research. Leadership Quart. **30**, 111-132.

[40] Daniell. 2018 Every Other Thursday: Stories and Strategies from Successful Women Scientists. New Haven, CT: Yale University Press.

[41] Mahmoudi M. 2020 A survivor's guide to academic bullying. Nat. Hum. Behav. **4**, Article 11. (10.1038/s41562-020-00937-1) 32868883

[42] Kording KP. 2021 For love of neuroscience: The Neuromatch movement. Neuron **109**, 3034-3035. (10.1016/j.neuron.2021.07.021) 34559980

[43] Stengers I. 2018 Another science is possible: a manifesto for slow science. Hoboken, NJ: John Wiley & Sons.

[44] Frith U. 2020 Fast lane to slow science. Trends Cogn. Sci. **24**, 1-2. (10.1016/j.tics.2019.10.007) 31744772

[45] Borck C. 2004 Message in a bottle from ‘the crisis of reality’: on Ludwig Fleck's interventions for an open epistemology. Stud. Hist. Philos. Sci. C **35**, 447-464.

[46] Rees T. 2016 Plastic reason: an anthropology of brain science in embryogenetic terms. Oakland: University of California Press.

[47] Dienes Z. 2021 The inner workings of Registered Reports. In Austin Lee Nichols & John E. Edlund (Eds), Cambridge Handbook of Research Methods and Statistics for the Social and Behavioral Sciences, Volume 2, See https://psyarxiv.com/yhp2a.

[48] Dienes Z. 2023 Testing theories with Bayes factors. In Austin Lee Nichols & John E. Edlund (Eds), Cambridge Handbook of Research Methods and Statistics for the Social and Behavioral Sciences, volume 1: Building a program of research, pp 494-512. Cambridge University Press. See https://psyarxiv.com/pxhd2.

[49] Landemore H. 2020 Open democracy: reinventing popular rule for the 21st century. Princeton, NJ: Princeton University Press.

[50] Dienes Z. 2022 The credibility crisis and democratic governance: How to reform university governance to be compatible with the nature of science. *R. Soc. Open Sci.* **10**, 220808. (http://doi.org/10.1098/rsos.220808). See https://psyarxiv.com/8wmna.

[51] Tiokhin L, Lakens D, Smaldino PE, Panchanathan K. 2021 Shifting the level of selection in science. *MetaArXiv Preprints*. (10.31222/osf.io/juwck) PMC11539478 37526118

[52] Praslova LN, Carucci R, Stokes C. 2022 How bullying manifests at work—and how to stop it. *Harvard Business Review*. See https://hbr.org/2022/11/how-bullying-manifests-at-work-and-how-to-stop-it (Accessed on 23 September 2023)

[53] Nik-Zainal S, Barroso I. 2019 Bullying investigations need a code of conduct. Nature **565**, 429. (10.1038/d41586-019-00228-4) 30675047

[54] Mahmoudi M. 2018 Improve reporting systems for academic bullying. Nature **562**, Article 7728. (10.1038/d41586-018-07154-x) 30356195

[55] Gewin V. 2021 How to blow the whistle on an academic bully. Nature **593**, Article 7858. 10.1038/d41586-021-01252-z 33976423

[56] Caron BR. 2020 Open Scientist Handbook. Version 2.0. Accessed on 1 December 2022. See https://openscientist.pubpub.org/pub/play/release/2

[57] Fortin JM, Currie DJ. 2013 Big science vs. little science: how scientific impact scales with funding. PLoS ONE **8**, e65263. (10.1371/journal.pone.0065263) 23840323 PMC3686789

